# The hospital autopsy: the importance in keeping autopsy an option

**DOI:** 10.4322/acr.2021.333

**Published:** 2022-02-17

**Authors:** Rachel Scarl, Bryce Parkinson, Vidya Arole, Tanner Hardy, Patricia Allenby

**Affiliations:** 1 The Ohio State University, Wexner Medical Center, Department of Pathology, Columbus, Ohio, USA

**Keywords:** Autopsy, COVID-19, SARS-CoV-2

## Abstract

Autopsy has been one of the most powerful diagnostic tools in medicine for over a century. Despite its importance in establishing cause of death and elucidating pathophysiology of disease, rates of hospital autopsies continue to decline. In this study we aim to determine if physicians believe autopsies are essential to patient care through discussion of autopsy with families. At the same time, we analyzed whether families are more willing to consent to autopsy if physicians are involved in autopsy discussion at the time of death, and what may be the reasons for not wanting an autopsy. Our results showed a doubling in autopsy consent when autopsy was discussed by the physician. Additionally, the biggest reason for families not consenting to autopsy was because they believed they already knew what caused death. The emergence of Coronavirus 2019 (COVID-19) has re-established the value of autopsy, as seen by increased autopsy rates in the past year. This study demonstrates that physician conversation with families on autopsy leads to an increased chance of autopsy consent.

## INTRODUCTION

For thousands of years, dissections of the human body occurred mostly for religious and cultural reasons. In more recent times, postmortem examinations became the cornerstone for understanding disease within the medical community.^1^ To this day, clinicians, students, and families can continue to benefit from what is discovered during the medical autopsy. On the medical practitioner side, autopsies can disclose whether a correct diagnosis was rendered, how the disease progressed and possibly discover new aspects of a disease not previously described. All of these aspects become important to provide quality assurance of care and continue to educate physicians on how diseases may be managed or cured.^2^

Additionally, students and physicians in training benefit from the practice of autopsy for a variety of reasons. First, performing autopsies allows not only the one doing the procedure to become proficient in anatomy but also becomes a valuable tool to teach other students. Through dissection of the body, students and residents can better understand disease progression and how it can lead to eventual death, an experience that simply cannot be read in a textbook. Learning how to perform an autopsy is likewise essential training for the pathology resident. If autopsies are not readily available to the pathology resident, the skills needed for postmortem examination decline, further making autopsy a less valuable tool. Finally, autopsies can help pathology residents learn to communicate findings to clinicians and enhance medical communication skills needed to become a successful pathologist.^3^

Families can also benefit from having a medical autopsy performed on a loved one. Autopsy allows a deeper exploration into what caused death in a patient which can be vitally important to bringing closure to families especially in instances of sudden or quick deaths. Postmortem examinations can also confirm that the patient received adequate care in the face of their illness and alleviate any guilt a family member might feel for not being able to do more. Finally, hereditable diseases discovered in the deceased otherwise unknown to family members provides benefit so early action and counseling can take place for those at risk.^2^

With the advancement of medicine including better diagnostic testing, advanced surgical procedures and higher quality imaging, the prospect of the autopsy providing additional information is becoming less clear in the minds of both clinicians and families alike. This is evident through the decrease in hospital autopsies, where autopsy rates in the 1940s were 50% or better compared to current autopsy rates hovering around 5%.^4^ Despite this decline, the recent severe acute respiratory syndrome coronavirus 2 (SARS-CoV-2), also known as the COVID-19 pandemic, reestablished the extreme importance of autopsy practices. Through examination of patients who succumbed to the disease, the medical community was able to learn more about the progression and thus provide more effective treatments to those suffering from severe disease. This profound example has demonstrated the importance of postmortem examinations and continuing to educate families and physicians of that importance is essential to keeping autopsy practices alive within the hospital setting. In this study, we examined whether autopsy is discussed by the clinician at the time of death of a patient with family members, and if so, why families decided not to proceed with the autopsy. Specifically, we wanted to identify if physician discussions about autopsy with the next of kin at the time of death correlated with a higher rate of consent for autopsy. Through this study, we hope to gain a better understanding of the perceptions of the medical autopsy and to reeducate the importance of postmortem examinations.

## METHODS

This study was conducted at The Ohio State University Wexner Medical Center (OSUWMC) and all data was collected via a SharePoint entry system within the electronic medical record system available at OSUWMC from June 2019 to July 2020. When a patient death occurred at the hospital, a SharePoint entry was created at the time of death, and this entry was updated as information about the decedent and their disposition became known. Specific data points were collected in this SharePoint system including name, medical record number (MRN), weight, date of birth, date of death, time of death, received date, received time, hospital unit, signing physician, Ohio State University (OSU) patient/non-OSU patient, Franklin County Corner notified, hold for autopsy, Lifeline of Ohio Procurement (LOOP) status release, funeral home, and next of kin with relationship and phone number.

To address the question of this study, three additional questions were added to the SharePoint entry system to determine if discussion about autopsy correlated with a consent for autopsy. These questions included: Did a physician discuss autopsy with the next of kin? Did the next of kin consent to an autopsy? If the next of kin declined the autopsy, what was the reason (if known)? The answers to these questions were compiled, along with demographic data of the decedent, and placed into an Excel document for further analysis and statistical comparison.

## RESULTS

A total of 1023 decedent data were collected for this study and defined based on whether autopsy was discussed with family (Autopsy Discussed) and whether the family consented to an autopsy (Autopsy Consented) (Table 1).

**Table 1:** Autopsy Discussion and Autopsy Consent: 2019-2020

		Autopsy Consented	
		No	Yes
Autopsy Discussed	No	464	30
	Yes	462	67

Of the 1023 deaths analyzed over June 2019 – July 2020, 494 cases did not involve autopsy discussion while 529 cases did. Of the 494 cases that did not involve autopsy discussion, 30 autopsy consents were obtained. Yet, of the 529 cases that did involve autopsy discussion, 67 autopsy consents were obtained. Therefore, a relationship between autopsy discussion and autopsy consent was determined (Chi squared = 12.179, df = 1, p-value = 0.0004834). In fact, as shown in Table 1, when the option for autopsy was discussed with families, there was a near doubling in the number of autopsies that were consented.

If autopsy discussions occurred and the family still opted to not consent for an autopsy, the reason behind not wanting an autopsy was also collected. The most common reason among families for not wanting to proceed with an autopsy was already knowing what caused the patient to die.

Other common reasons included that the decedent would have never wanted an autopsy, the decedent had been through enough already, and the family was too emotionally distraught to consider an autopsy at the time. Less commonly, some families did not consider autopsy for religious purposes (Orthodox Jewish, Muslim) or because the decedent was a ward of the state.

Data that were collected during the 2019 – 2020 academic year was further divided to demonstrate trends of autopsy discussion versus autopsy consent before the COVID-19 pandemic (Table 2, defined as June 2019 – March 2020) and compared to trends during the coronavirus 2019 pandemic (Table 3, defined as April 2020 – July 2020). Of the 793 deaths analyzed over June 2019 – March 2020 (Table 2), 355 cases did not involve autopsy discussion leading to 26 consents obtained while 367 cases did involve discussions leading to 45 autopsy consents (Chi-squared = 3.5911, df = 1, p-value = 0.05809).

**Table 2:** Autopsy Discussion and Autopsy Consent Before COVID-19

		Autopsy Consent	
		No	Yes
Autopsy Discussed	No	355	26
	Yes	367	45

**Table 3:** Autopsy Discussion and Autopsy Consent During COVID-19

		Autopsy Consent	
		No	Yes
Autopsy Discussed	No	109	4
	Yes	95	22

Interesting, of the 230 deaths analyzed over April 2020 – June 2020 (Table 3), a stronger relationship between autopsy discussions and autopsy consent was seen (Chi-squared = 11.878, df = 1, p-value = 0.000568). Comparatively, before COVID-19, approximately 11% of autopsies were consented if discussions occurred by the physician, whereas during the COVID-19 pandemic, approximately 19% of autopsies were consented if discussions occurred by the physician.

## DISCUSSION

Through our study, we have determined that autopsies consented rely on both family understanding and physician belief of its importance. The reasoning for why the next of kin would decline an autopsy can be broad. Most common reasons for declining an autopsy are stress around the time of death, lack of rapport with physicians, inadequate information about the value of an autopsy, concerns about disfigurement of a loved one, concerns about delayed funeral services, concerns about cost, and religious objections.^5^ Our study showed the most common causes for declining an autopsy were: the family already knew the cause of death, the decedent would not have wanted an autopsy performed, the decedent had already been through enough, and the family felt too much emotional distress to consider an autopsy. The least common reason in our study was denial due to religious preferences in the Muslim or Orthodox Jewish community.

Our most common reason for a declined autopsy was the family already knew the cause of death. A common belief is that an autopsy will not provide additional information once the pre-mortem cause of death has already been established. The advent and development of less-invasive medical technology, and increased diagnostic capacity through imaging studies and laboratory tests, has been offered as a leading reason for the decline in hospital autopsies. With the supposed and expected diagnostic capacity and confidence, in the majority of cases, people feel an autopsy will not provide any additional information that was not already known at the time of death.^6^^,^ 7 Despite the increase in medical technology, autopsies have identified and documented various and significant diagnostic discrepancies when comparing the antemortem diagnosis to the postmortem diagnosis, and when evaluating for class 1 and 2 errors.^4^^,^ 8

In 2003, a systematic literature review identified 45 studies from 1966 to 2002, that evaluated 53 distinct autopsy series for the presence of major diagnostic errors (defined as clinically missed diagnoses involving a primary cause of death) and class 1 errors.^8^ Class 1 errors are defined as the rejection of a true null hypothesis which is also knows as a false positive, and in this study was related patient outcomes. Of these 53 autopsy series, 42 reported major diagnostic errors, 37 reported class 1 errors, and 26 autopsy series reported both major errors and class 1 errors. Overall, the median major error rate was 23.5% and the median class 1 error rate was 9.0%. Furthermore, they estimated that at a contemporary US institution, where the autopsy rate is approximately 5%, they can expect a major error rate ranging from 8.4% to 24.4%, and a class 1 error rate from 4.1% to 6.7%.^8^

In addition, a 2007 retrospective review of medical records and autopsy records was performed to identify all cancer patients who died in a medical-surgical ICU and had an autopsy performed, between 1 January 1999 and 30 September 2005.^9^ The postmortem clinical diagnoses were compared with the postmortem findings identified after autopsy. Of the 86 patients they evaluated, 22 (26%) had major missed diagnoses, 12 (54%) had class 1 discrepancies, 7 (32%) had class 2 discrepancies, and 3 (14%) had both class 1 and class 2 discrepancies. They identified an overall discrepancy rate of 26% between a patient’s antemortem clinical diagnosis and the postmortem diagnosis in this group of cancer patients.^9^

An overconfidence in the accuracy of diagnostic medical technology may play a role in discrediting the role autopsies play in providing diagnostic information. Although studies have identified this concern and highlight the vital role an autopsy still plays in the current medical environment.^8^^,^ 9 Thus, if autopsies are used properly, this valuable tool can continue to add important information to our diagnostic process. Discussing the benefits of an autopsy may help families understand the vital role their loved one plays in the medical community, and the important information we can glean regarding disease processes, treatment efficacy, diagnostic approaches, prevention of medical errors, and public health information.

The religious beliefs of a decedent/next-of-kin also plays a role in influencing autopsy rates. The burial process, and honorance of the deceased, is a storied and cherished tradition in most world religions and can widely vary from one religion to another. Most religions permit the autopsy for the purposes of instruction, scientific research, and the pursuit of justice.^5^ The role of the pathologists encompasses the instruction and reassure the next-of-kin that an autopsy (1) does not disfigure or mutilate the deceased (2) maintains and preserves the decedent’s respect and honor (3) will not delay funeral proceedings or the ability to hold an open-casket funeral viewing and (4) can serve the medical/scientific community by providing vital medical information.

It is also important that we understand why physicians are or are not discussing autopsy with the next of kin and how we can improve motivation in physicians to discuss autopsy. Autopsy rates in hospitals of the United States have decreased significantly over the past 50 years and physicians play a key role in influencing whether an autopsy is performed, as shown above. Therefore, understanding motivations for or against autopsy from physicians is critical. One study performed by Burton et al.^10^ noted the autopsy rate had decreased from 60% in the 1950s to less than 6% in the early 2000s. In their study they found that if physicians gave a strong recommendation for autopsy that they were more likely to be performed as opposed to a weak recommendation or no recommendation. Reasons why physicians might not recommend autopsy include belief that there is no new knowledge to be gained regarding the cause of death, desire to avoid additional patient and/or family suffering, no contributions to the scientific community could be made, the cost and/or lack of reimbursement for autopsy, and a possible increase in the likelihood of malpractice litigation involved in inappropriate patient care.^8^^,^ 11 ^,^ 12 Therefore, many physicians today have lost interest in autopsy in favor of molecular tests and/or imaging to arrive at a conclusion pertaining to cause of death.^8^

Pathologists themselves may also be to blame in the decline of autopsy rates. A small survey recently highlighted pathology resident’s views of the hospital autopsy in which a majority of those surveyed did not view autopsy as beneficial to their training (mean score of 3.6 out of 10, with 10 being most important).^13^ Recent changes to board certification for pathology residents included a decreased number of required autopsies from 50 to 30, raising questions whether this will further decrease interest in performing autopsy or provide the experience needed to be competent in the practice.^13^ Additionally, pathology faculty have also been shown to have little interest in autopsy or little experience in overseeing and teaching autopsy, demonstrating that the importance of autopsy is lost further with each new generation of pathologists.^14^

Further studies are needed to show how we can improve physician understanding of autopsy and increase discussions with next of kin regarding them. However, we propose that if physicians were educated regarding the benefits of autopsy through seminars conducted by pathologists, for example, that there would be greater motivation to discuss and recommend autopsy. For instance, exposing physicians in training, such as medical students and residents, to actual autopsy cases with specific findings that may differ from initial clinical impression emphasizes the importance of autopsy early in clinical training. Autopsy provides further insight to the cause of death, which is sometimes not appreciated with laboratory tests and imaging. There is also benefit to the scientific community. For example, autopsy has played a role in our understanding of the effects of COVID-19 in the course of the disease. When autopsy results do not correlate well with the clinical impression of cause of death there is opportunity to learn. To address fear of increased malpractice lawsuits, studies have demonstrated no relationship between performing an autopsy and increased unfavorable outcomes in defendant physician malpractice litigations.^11^ In fact, there is evidence of more outcomes favoring defendant physicians rather than favoring the plaintiff when an autopsy is involved.^11^ Studies need to be performed to determine what means are the best to educate physicians regarding autopsy. Such methods could include regularly scheduled seminars between clinicians and pathologists, improvement of autopsy rooms to involve more hands-on teaching of medical students and residents from all specialties, improved safety equipment especially in cases of infectious disease, modular learning, or virtual discussions. In fact, reminding clinicians of the importance of clinicopathological correlations’ that is further elucidated in autopsy continues to improve care for future patients especially in the age of understanding the human genome.^15^

In the wake of a worldwide pandemic beginning in 2020 in the United States, the importance of the hospital autopsy was reinvigorated in the minds of both clinicians and families alike. As the country-wide hospital system continued to battle against a novel virus, physicians searched for therapies and a better understanding of how the virus affected the body. Although imaging and other diagnostic testing were helpful, it was the autopsy that could provide ultimate clarity into the pathophysiology of this infection. Despite initial concerns about infectivity, the rates of hospital autopsies began to rise as pathologists, clinicians and families searched for answers to the unknown. At New Orleans University Medical Center, for example, pathologists have performed about 50% more autopsies than they have in recent years. Even at OSUWMC, an increase in autopsy consents was seen from 11% consented autopsies before COVID-19 pandemic to 19% consented autopsies in the midst of the COVID-19 pandemic. Aside from providing new insight into a novel disease, this jump in autopsy consent likely resulted from increased time to consider autopsy. Specifically, at OSUWMC, visitations by families were highly restricted in the wake of the pandemic, therefore, extensive discussions by physicians and autopsy administrative staff did not occur. This likely led to lack of understanding since families could not be present during the course of the hospital stay and identify what may have ultimately led to death. Additionally, without discussions led by autopsy administrative staff, families had more time to decide on whether to proceed with an autopsy. It is also worth noting that autopsy consent in the non-discussed group during COVID-19 (3.5%, Table 3) was almost half of the consent in non-discussed group before COVID-19 (6.8%, Table 2) which likely is an effect of the lack of face-to-face communication that occurred during the pandemic between autopsy staff and families in the education of autopsy.

The importance of autopsy was thus reestablished. Early autopsies of deceased patients demonstrated that the virus is not limited to respiratory disease but can also attack other vital organs. With new and better understanding of this virus, physicians began to try blood thinners in COVID-19 patients and determine how long others should be on ventilators. All these therapies only became established after autopsy could share answers, therapies that could further save millions of lives. Thus, the COVID-19 pandemic demonstrated the vital importance of not only understanding this novel virus but also gaining insight to lifesaving treatments and therapies.

## CONCLUSION

As medicine continues to advance with new technology, diagnostic modalities, and treatments, it is important to remember the cornerstone on which diseases were first witness and understood. Even with pathologies that are well-known, we can still gleam valuable medical knowledge from autopsies that show these pathologies. Together with clinicians and families, remembering the important value of autopsy will continue to shape our medical community and allow for advancements in therapy and treatment.
